# Obesity and Early-Onset Breast Cancer and Specific Molecular Subtype Diagnosis in Black and White Women: NIMHD Social Epigenomics Program

**DOI:** 10.1001/jamanetworkopen.2024.21846

**Published:** 2024-07-29

**Authors:** Sarabjeet Kour Sudan, Amod Sharma, Kunwar Somesh Vikramdeo, Wade Davis, Sachin K. Deshmukh, Teja Poosarla, Nicolette P. Holliday, Pranitha Prodduturvar, Cindy Nelson, Karan P. Singh, Ajay P. Singh, Seema Singh

**Affiliations:** 1 Cancer Biology Program, Mitchell Cancer Institute, University of South Alabama, Mobile; 2 Department of Pathology, Frederick P. Whiddon College of Medicine, University of South Alabama, Mobile; 3 Department of Cell and Molecular Biology and Cancer Center and Research Institute, University of Mississippi Medical Center, Jackson, Mississippi; 4 Interdisciplinary Clinical Oncology, Mitchell Cancer Institute, University of South Alabama, Mobile; 5 Department of Obstetrics and Gynecology, Frederick P. Whiddon College of Medicine, University of South Alabama, Mobile; 6 Department of Epidemiology & Biostatistics, School of Medicine, University of Texas Health Science Center at Tyler, Tyler; 7 Department of Biochemistry and Molecular Biology, Frederick P. Whiddon College of Medicine, University of South Alabama, Mobile

## Abstract

**Importance:**

Epidemiologic data suggest an association of obesity with breast cancer (BC); however, obesity’s contribution to early onset and risk of diagnosis with specific molecular subtypes by race is uncertain.

**Objective:**

To examine the race-specific association of body mass index with early onset and diagnosis of specific molecular subtypes.

**Design, Setting, and Participants:**

This retrospective cohort study included patients with BC diagnosed between October 1, 2017, and March 31, 2022, at 3 University of South Alabama Mitchell Cancer Institute clinics. Participants were also prospectively enrolled for serum leptin measurement.

**Main Outcomes and Measures:**

The primary outcome was age at BC onset and specific subtype diagnosis. The secondary outcome was race-specific differences. Odds ratios (ORs) for associations of body mass index with age at onset and subtype were estimated using the Fisher exact test. Race was self-reported.

**Results:**

Of the 1085 study patients, 332 (30.6%) were Black with a median age of 58 (IQR, 50-66) years, and 753 (69.4%) were White with a median age of 63 (IQR, 53-71) years. A total of 499 patients (46.0%) had obesity, with Black women with obesity receiving more frequent BC diagnosis than their White counterparts (OR, 2.40; 95% CI, 1.87-3.15; *P* < .001). In addition, Black women had a significantly higher incidence of early-onset disease (OR, 1.95; 95% CI, 1.33-2.86; *P* = .001) than White women, and obesity increased this risk significantly in Black women (OR, 2.92; 95% CI, 1.35-6.22; *P* = .006). Black women with obesity also had a significantly higher risk of luminal A BC (OR, 2.53; 95% CI, 1.81-3.56; *P* < .001) and triple-negative BC (TNBC) (OR, 2.48; 95% CI, 1.43-4.22; *P* = .002) diagnosis than White counterparts. Black women, with or without BC, had significantly higher serum leptin levels (median [IQR], 55.3 [40.3-66.2] ng/mL and 29.1 [21.1-46.5] ng/mL, respectively, *P* < .001) than White women (median [IQR], 33.4 [18.9-47.7] ng/mL and 16.5 [10.0-22.9] ng/mL, respectively), which was associated with higher odds of luminal A disease (OR, 5.25; 95% CI, 1.69-14.32, *P* = .003). Higher odds of early-onset disease (OR, 3.50; 95% CI, 0.43-23.15; *P* = .33 for trend), and TNBC diagnosis (OR, 6.00; 95% CI, 0.83-37.27; *P* = .14 for trend) were also seen, although these outcomes were not statistically significant.

**Conclusions and Relevance:**

In this cohort study of patients with BC, obesity and high serum leptin levels were associated with an enhanced risk of early-onset BC and diagnosis of luminal A and TNBC subtypes in Black women. These findings should help in developing strategies to narrow the existing disparity gaps.

## Key Points

**Question:**

Does obesity have an association with early-onset breast cancer and diagnosis of specific molecular subtypes?

**Findings:**

In this cohort study of 1085 patients with breast cancer, those with obesity and of Black race were diagnosed more often with breast cancer than their White counterparts. Furthermore, Black women with obesity exhibited a significantly higher risk of early onset and diagnosis of luminal A and triple-negative breast cancer subtypes.

**Meaning:**

Findings suggest that obesity is a significant predisposing health condition associated with observed disparities in age of breast cancer onset and is also a risk factor for diagnosis of luminal A and triple-negative breast cancer molecular subtypes.

## Teaser

This cohort study of patients with breast cancer examines whether a race-specific association exists between obesity and early-onset breast cancer or the diagnosis of specific molecular subtypes.

## Introduction

Obesity, defined as the excessive accumulation of fat, is a concerning disorder that increases the risk of various other health problems. Black women in the US tend to have more obesity based on body mass index (BMI) than their non-Hispanic White counterparts.^1,2^ Social, economic, psychological, and neighborhood factors likely contribute to this racial disparity, along with increasing the risk of several other diseases, including cancer.^1,3,4^ Breast cancer (BC) is the most commonly diagnosed cancer and the second leading cause of cancer-related death among US women. It also exhibits significant race-associated health disparities.^5,6^ Although both Black and White women have nearly equal rates of BC diagnosis, the incidence of BC diagnosis in individuals younger than 45 years is higher among Black women.^7,8^ Black women are also more likely to be diagnosed with an aggressive molecular subtype, triple-negative BC (TNBC or basal-like), compared with their White counterparts.^9,10^ In addition, Black women with hormone receptor–positive, *ERBB2* (OMIM 164870) (previously *HER2*/*neu*)–negative BC (luminal A subtype) experience a high disparity in survival, contributing to their overall higher mortality.^11^

Leptin is an obesity-associated molecule functioning both as a hormone and a cytokine. It is known to have antiapoptotic, mitotic, and proangiogenic roles through direct actions via its receptor (Ob-R) and its crosstalk with other oncogenic signaling pathways.^12,13,14,15,16^ Thus, leptin could be an important part of the nexus of social experiences, obesity, and race-associated BC disparities. Several race-associated differences in BC biology have been reported. *SOS1* (OMIM 182530), a key regulator of Ras signaling, is overexpressed in BC in Black women compared with White women. It is shown to promote metastasis through the obesity-activated hepatocyte growth factor receptor pathway.^17^ Likewise, a role of cancer disparity-linked genes, *CRYBB2* and *CRYBB2P1* (OMIM 123620), has been reported in BC.^18^ Other genetic differences, specifically those pertaining to immune responses, have also been reported and suggested to have an association with higher rates of TNBC in Black women.^19^

In this exploratory cohort study, we sought to identify whether obesity has an association with early-onset BC in Black women and favors diagnosis with a specific molecular subtype, such as TNBC. We also analyzed whether levels of leptin in a patient’s serum vary by race and exhibit a similar association to suggest its potential as a biological risk factor and mediator of pathobiological association.

## Methods

### Study Population

For the current retrospective cohort study, clinical records of patients between October 1, 2017, and March 31, 2022, were pulled from 3 University of South Alabama Mitchell Cancer Institute (MCI) clinics (MCI Mobile–Children’s and Women’s Hospital, MCI Fairhope–University Hospital, and MCI Springhill–University Hospital) with geographic bounds concentrated in a single metropolitan area. Details of the number of patients with their counties and states are given in eTable 1 in Supplement 1. Women who self-reported as Black or White and had confirmed BC diagnosis with no prior history of any other cancer and who were older than 18 years at the time of diagnosis were included. Other inclusion criteria were available information on BMI and BC molecular subtypes. Black and White women with or without BC diagnosis were also prospectively recruited for blood biomarker analysis. Written informed consent was obtained from all the volunteers, and the collected data were deidentified. The study protocol was reviewed and approved by the USA Institutional Review Board, and all the relevant regulations and guidelines were followed. We followed the Strengthening the Reporting of Observational Studies in Epidemiology (STROBE) reporting guideline.^20^

### Definition of Obesity, Early Onset, and BC Molecular Subtypes

The World Health Organization defines obesity as excessive fat accumulation posing a risk to health. A person with a BMI (calculated as weight in kilograms divided by height in meters squared) of 30 or higher is considered to have obesity; between 25 and 30, overweight; and less than 25, normal weight. Body mass index was recorded at patients’ visits to the clinic before any surgery. Early-onset BC is defined as a diagnosis made before age 45 years.^21^ For association analysis, we divided the patients into 3 age categories (<45, 45-65, and >65 years). On the basis of the expression of hormone receptors (HRs), which included estrogen receptors (ERs) and progesterone receptors (PRs) and *ERBB2* receptor, BC cases were classified into 4 molecular subtypes: luminal A (HR positive and *ERBB2* negative), luminal B (HR positive and *ERBB2* positive), *ERBB2* enriched (HR negative and *ERBB2* positive), and basal BC or TNBC (HR negative and *ERBB2* negative). Patients with HR-positive BC were positive for ER, PR, or both.

### Leptin: An Adiposity Biomarker

Leptin is a hormone predominantly produced by adipocytes and is regarded as a biomarker of fat mass.^22^ Its primary role is to regulate appetite and energy balance, but it can also influence several other biological processes, including carcinogenesis.^23,24^ Serum leptin levels were measured in prospectively enrolled patients with BC or non-BC diagnosis using a commercial enzyme-linked immunosorbent assay kit (R&D Systems) as per the manufacturer’s instructions. On the basis of the median of leptin levels, participants were divided into 3 categories: low, when levels were lower than the median level (<21.9 ng/mL) in women without BC; high, when above the median level (>44.1 ng/mL) in women with BC; and moderate, when the levels were in between (21.9-44.1 ng/mL).

### Statistical Analysis

Patients were compared based on their BMI, age at BC onset, and molecular subtypes. The descriptive statistics were computed to calculate the mean (SD) and median (IQR). The Fisher exact test was used to calculate the odds ratio (ORs) and 95% CIs for Black vs White women with respect to categorized age, BMI, and BC subtypes. Serum leptin levels were measured in triplicates, and the 2-tailed, unpaired *t* test was performed to compare the mean levels in BC and non-BC (control) groups. Pearson correlation analysis was performed to study the linear association between BMI and leptin levels. Results were considered significant with a 2-sided *P* < .05. All statistical calculations were performed using GraphPad Prism, version 8 (GraphPad Software Inc).

## Results

**Figure 1.**
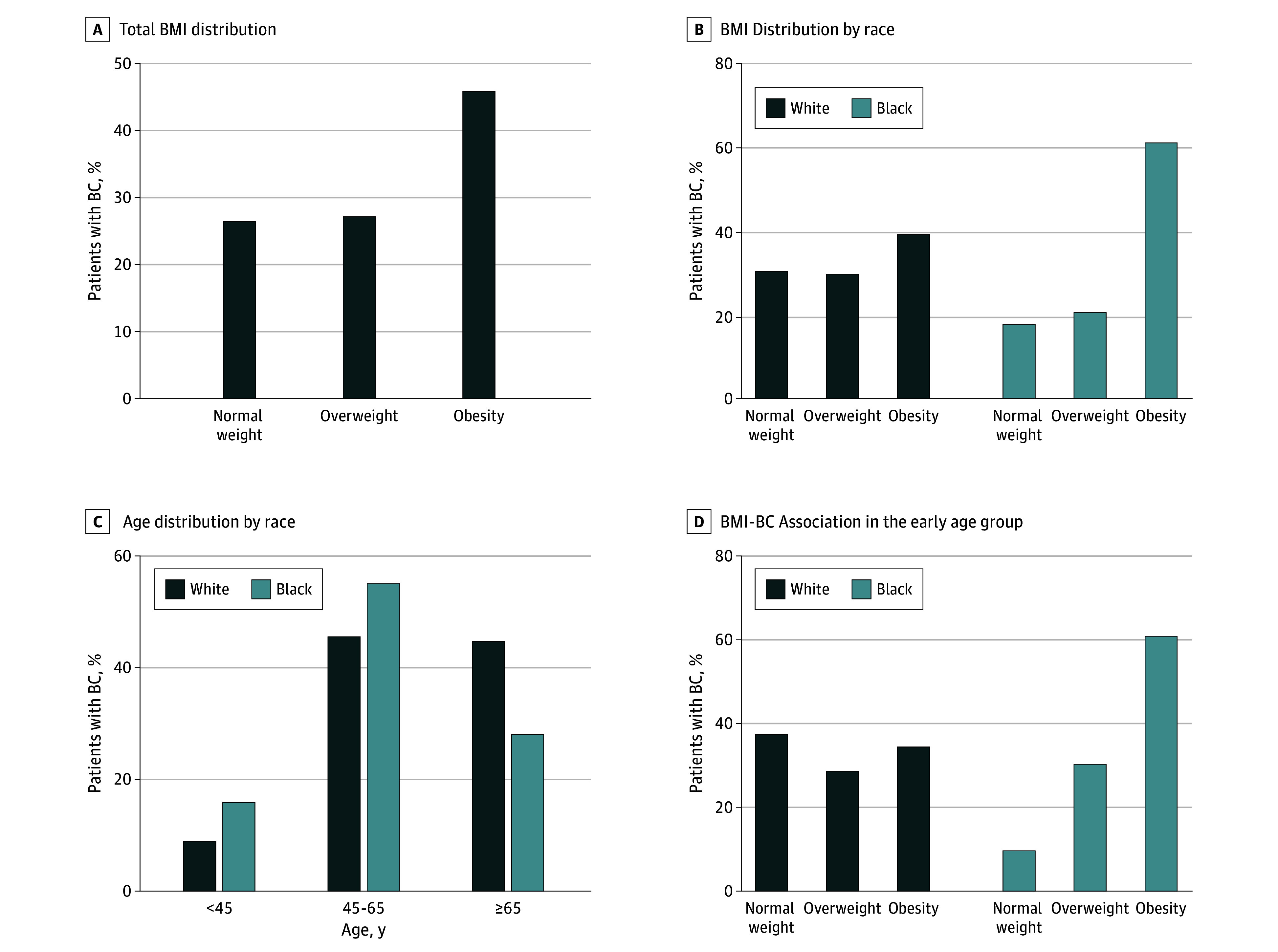
Association of Breast Cancer (BC) With Body Mass Index (BMI) and Age A, Distribution of BMI (calculated as weight in kilograms divided by height in meters squared) in patients with BC (n = 1085). All patients were divided into 3 BMI categories: less than 24.9 (normal weight), 25.0 to 29.9 (overweight), and greater than 30.0 (obesity). B, Distribution of BMI in patients with BC by race. C, Distribution of age between Black and White women. Total patients were divided into 3 age categories: younger than 45, 45 to 65, and older than 65 years. D, Association of BMI with BC among the early age group (aged <45 years) between Black and White women.

We included a cohort of 1085 women with a confirmed BC diagnosis. Among them, 332 (30.6%) were Black and 753 (69.4%) were White based on self-reporting. Median (IQR) age at diagnosis was 58 (50-66) in the Black women and 63 (53-71) years in the White women. The median (IQR) BMI was 32.5 (27.5-38.0) in the Black women and 28.2 (24.0-32.7) in the White women (Table). Among the 3 categories of BMI, the highest percentage of women with BC fell in the obesity category (499 [46.0%]), with nearly equal distribution in the normal weight and overweight groups (Figure 1A). However, Black women with obesity were at a significantly higher risk of being diagnosed with BC than White women (OR, 2.40; 95% CI, 1.87-3.15; *P* < .001) (Figure 1B). Significant differences in the age distribution of patients with BC were also reported, with Black women exhibiting a higher incidence of early-onset BC (age <45 years) compared with White women. Black women were 1.95 times more likely to be diagnosed with BC than White women at an early age (95% CI, 1.33-2.86; *P* = .001) (Figure 1C). Notably, Black women also exhibited a significantly positive association of obesity with early-onset BC (OR, 2.92; 95% CI, 1.35-6.22; *P* = .006) (Figure 1D).

**Table.** Baseline Characteristics of the Retrospective Cohort (2017-2022)^a^

Characteristic	Black	White
Total patients	332 (30.6)	753 (69.4)
Age, median (IQR), y	58 (50-66)	63 (53-71)
BMI, median (IQR)	32.5 (27.5-38.0)	28.2 (24.0-32.7)
BMI categories		
Normal weight	60 (18.1)	230 (30.5)
Overweight	69 (20.8)	227 (30.2)
Obesity	203 (61.1)	296 (39.3)
Subtype		
Luminal A	191 (57.5)	547 (72.6)
Luminal B	25 (7.5)	67 (8.9)
*ERBB2* enriched	10 (3.0)	34 (4.5)
TNBC	106 (32.0)	105 (14.0)

Abbreviations: BMI, body mass index (calculated as weight in kilograms divided by height in meters squared); TNBC, triple-negative breast cancer.

^a^ Data are presented as number (percentage) of patients unless otherwise indicated.

**Figure 2.**
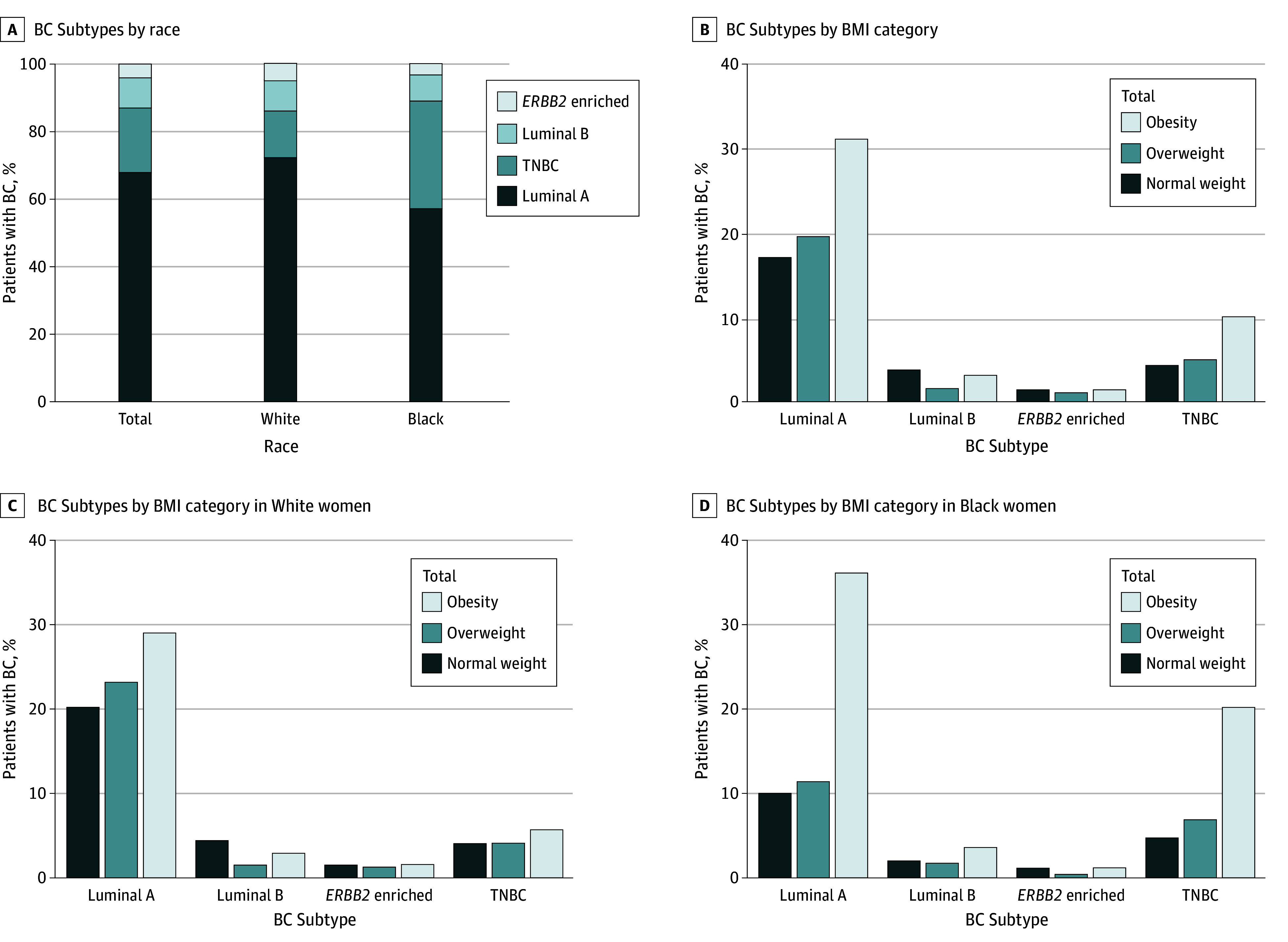
Prevalence of Breast Cancer (BC) Subtypes in Association With Body Mass Index (BMI) A, Percentage distribution of BC subtypes from a retrospective cohort among total, Black, and White women. B, Percentage distribution of BC subtypes under 3 BMI (calculated as weight in kilograms divided by height in meters squared) groups in the total cohort of patients. C and D, Percentage distribution of BC subtypes under 3 BMI groups between White (C) and Black (D) women. TNBC indicates triple-negative BC.

All patients were most frequently diagnosed with the luminal A subtype (Black, 191 [57.5%]; White, 547 [72.6%]), followed by TNBC (Black, 106 [32.0]%; White, 105 [14.0%]), luminal B (Black, 25 [7.5%]; White, 67 [8.9%]), and *ERBB2*-enriched (Black, 10 [3.0%]; White, 34 [4.5%]) subtypes. Black women were more likely to receive a TNBC diagnosis than White women (OR, 2.90; 95% CI, 2.12-3.96; *P* < .001), whereas White women were more likely to be diagnosed with the luminal A subtype compared with Black women (OR, 1.96; 95% CI, 1.50-2.56; *P* < .001) (Figure 2A). A positive association of obesity with the diagnosis of TNBC subtype (OR, 1.36; 95% CI, 1.01-1.83; *P* = .05 for trend) was observed. Higher odds of diagnosis of luminal A subtype were also observed, although this finding was not statistically significant (OR, 1.17; 95% CI, 0.90-1.57; *P* = .26 for trend) (Figure 2B). Moreover, the odds for Black women with obesity were 153% and 148% higher compared with White women with obesity for the diagnosis of luminal A BC (OR, 2.53; 95% CI, 1.81-3.56; *P* < .001) and TNBC (OR, 2.48; 95% CI, 1.43-4.22; *P* = .002), respectively (Figure 2C and D).

**Figure 3.**
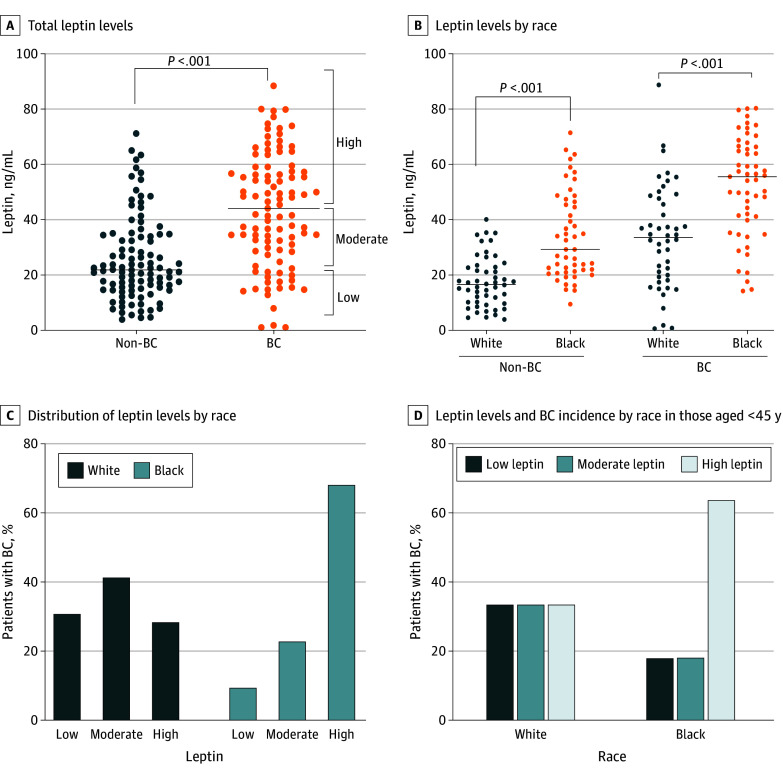
Levels of Serum Leptin and Association With Early-Onset Breast Cancer (BC) A, Serum levels of leptin in patients without BC (n = 100) and patients with BC (n = 99). B, Serum levels of leptin in Black and White women (n = 50 each for non-BC group and n = 53 and 46 for the BC groups, respectively. C, Distribution of leptin levels between Black and White women in patients with BC. D, Association of leptin levels with BC incidence among the early age group (aged <45 years) between Black and White women.

**Figure 4.**
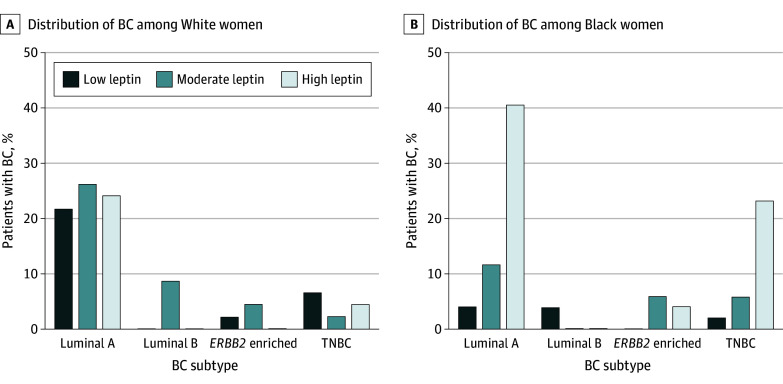
Association of Serum Leptin Levels With Breast Cancer (BC) Subtype Diagnosis Percentage distribution of BC subtypes among 3 leptin groups between White and Black women. TNBC indicates triple-negative BC.

To observe whether leptin, being a bioactive signaling molecule, could mediate the risk association of BMI with early-onset BC and luminal A and TNBC diagnosis, we prospectively enrolled 99 patients with BC (53 Black and 46 White) along with 100 controls without BC (50 each from self-reported Black and White racial groups) (eTable 2 in Supplement 1). We observed significantly elevated serum leptin levels in women with a BC diagnosis (median [IQR], 44.1 [28.6-57.4] ng/mL) compared with the control group (median [IQR], 21.9 [15.2-34.3] ng/mL) (*P* < .001) (Figure 3A). Notably, Black women with or without BC exhibited significantly higher leptin levels (median [IQR], 55.3 [40.3-66.2] ng/mL and 29.1 [21.1-46.5] ng/mL, respectively) than White women (median [IQR], 33.4 [18.9-47.7] ng/mL and 16.5 [10.0-22.9] ng/mL, respectively) (*P* < .001) (Figure 3B; eTable 3 in Supplement 1). Leptin levels were positively correlated with BMI in both patients with BC (*r* = 0.52, *P* < .001) and control patients (*r* = 0.55, *P* < .001) in both racial groups; however, a slightly stronger association was observed in Black patients with BC (*r* = 0.48, *P* < .001) or Black controls without BC (*r* = 0.56, *P* < .001) compared with their White counterparts (*r* = 0.46, *P* = .001 for both groups) (eFigure in Supplement 1). Black women with high leptin levels were more likely to be diagnosed with BC than White women (OR, 5.38; 95% CI, 2.18-12.35; *P* < .001) (Figure 3C). Furthermore, compared with White women, we observed higher odds of early-onset BC in Black women, although this finding was not statistically significant (OR, 3.50; 95% CI, 0.43-23.15; *P* = .33 for trend) (Figure 3D). In addition, higher leptin levels were associated with increased odds of being diagnosed with luminal A (OR, 5.25; 95% CI, 1.69-14.32; *P* = .003) in Black women compared with White women. However, there was no statistically significant difference for TNBC diagnosis (OR, 6.00; 95% CI, 0.83-37.27; *P* = .14 for trend) (Figure 4).

## Discussion

Obesity plays a significant role in the development of cancer. Data presented in this study provide novel evidence suggesting an association of obesity with early-onset BC and the risk of diagnosis with luminal A and TNBC molecular subtypes, with a higher risk in Black women. The global epidemic of obesity is continuously increasing; however, a disparate prevalence of obesity exists among different racial groups. The prevalence of obesity in Black women is 56.9% compared with 39.8% in White women in the US.^25^ Furthermore, Black women, although exhibiting a similar incidence rate for BC diagnosis as their White counterparts, are more often diagnosed at a younger age. Black women also present with more aggressive disease and have a poorer response to existing therapies, which contributes to their overall higher mortality.^10,26^

While analyzing the retrospective cohort dataset, we found that most women with BC had a BMI that fell in the obesity category. Furthermore, in a race-wise comparison, we observed that the percentage of Black women with obesity was significantly higher than that of White women of a similar BMI. As previously reported,^8^ we also observed a higher incidence of early-onset BC in Black women in our cohort. Interestingly, when we compared BMI distribution in early onset of BC, we found that Black women with obesity were at a significantly higher risk compared with White women. These observations suggest that although increased BMI may be a predominant risk factor associated with the diagnosis of BC at a younger age in Black women, early onset of BC in White women likely results from genetic predisposition or other risk behaviors. Among the genetic risk factors, *BRCA1* (OMIM 113705) and *BRCA2* (OMIM 600185) are most commonly (>50%) associated with early-onset BC.^21^ Some race-associated differences in the frequency of these mutations are observed, with Black women having a lower frequency of *BRCA1* and a relatively higher frequency of *BRCA2* than White women.^21^

In our study, we also found that a higher percentage of patients with increasing BMI were diagnosed with luminal A and TNBC subtypes in both Black and White women; however, the risk was significantly higher in Black women for both the molecular subtypes. A previous study found that higher adiposity was associated with a risk of BC development in postmenopausal women, especially the risk of being diagnosed with HR-positive but not HR-negative BC.^27^ In addition, women with obesity are more likely to have lymph node metastasis associated with poorer survival.^28,29^ Furthermore, obesity can impact the treatment response, including endocrine therapy primarily administered to patients with HR-positive BC.^30^ A previous study also found that obesity increased the risk of recurrence among patients with HR-positive BC treated with aromatase inhibitors.^28^ Thus, a higher rate of obesity in Black women with HR-positive BC could be associated with disparate high mortality rates for this subtype. Triple-negative BC is a highly aggressive molecular subtype that is diagnosed more frequently in Black women and has limited available therapeutic options.^31,32^ Patients with TNBC often have their disease diagnosed at higher stages and grades and often have large, fast-growing tumors. Earlier studies have demonstrated that obesity is positively associated with increasing stage and grade of tumor in patients with TNBC.^33,34,35^ Thus, it appears that obesity and race should be evaluated as a significant risk factor for the diagnosis of aggressive disease molecular subtypes as well as the disease outcomes.

Leptin is a signaling hormone produced by adipose cells and has been shown to have a causative link with BC.^36^ It not only enhances the progression of HR-positive BC also promotes chemoresistance in TNBC cells.^37,38^ We found that leptin levels were increased in the serum of patients with BC. Furthermore, Black women exhibited higher serum levels of leptin than White women regardless of their BC diagnosis. Furthermore, leptin levels were positively associated with the patient’s BMI and exhibited a similar association with age at disease onset and molecular subtype diagnosis. These data suggest that leptin could be a biological intermediary for the observed association of obesity with early onset of BC and specific subtype diagnosis. However, because we observed a significantly stronger association in Black patients, other race-associated risk factor(s) should be analyzed that likely work in concert with leptin at the signaling level to support early development of BC and favor the growth of luminal A and TNBC molecular subtypes. A previous report highlighted the differences in the African ancestry–associated genes in patients with TNBC that impact tumor biology and clinical outcomes.^39^ Thus, it will be interesting to see whether leptin signaling has a molecular crosstalk with these African ancestry–associated genes and whether that contributes to observed disparities in age at onset and more frequent diagnosis of luminal A and TNBC subtypes in Black women with obesity.

### Limitations

This study has several limitations. First, this is a single-institution study enrolling patients who visited clinics during 5 years. Information on BC subtype for all patients was difficult to access, especially those who visited clinics before 2017, due to changes in the electronic medical record management server that resulted in a small sample size. In addition, despite our best efforts, it was not possible to enroll a large cohort of patients in the prospective study. Second, racial grouping was solely based on self-reporting; thus, there is a risk of misrepresentation.

## Conclusions

Our findings suggest that obesity is associated with an early onset of BC and a higher risk of diagnosis with luminal A or TNBC subtypes, especially in Black women. In addition, higher levels of serum leptin appear to be an important biological intermediary in these risk associations. Additional race-associated risk factors should be explored, such as social exposures and economic and psychological stressors that promote obesity rates in Black women and may have a broader effect on human biology. These factors may create further hormonal imbalance or inflammation that may lead to aggressive progression of breast tumors in Black women, leading to its presentation at an early age. Future research along these lines could increase our understanding of the underlying causes of BC racial disparities and help shape community-based approaches to diminish the existing disparity gaps.

## Notes

Supplement 1. **eFigure.** Correlation Between Serum Leptin Levels and BMI **eTable 1.** Details of Counties and States of Retrospective Cohort (2017-2022) **eTable 2.** Characteristics of Prospectively Enrolled Patients **eTable 3.** Serum Levels of Leptin in BC and Non-BC Patients

Supplement 2.
Data Sharing Statement
