# Supplementary material for: Obesity and Early-Onset Breast Cancer and Specific Molecular Subtype Diagnosis in Black and White Women: NIMHD Social Epigenomics Program
Source: JAMA Netw Open. 2024 Jul 29;7(7):e2421846. doi: 10.1001/jamanetworkopen.2024.21846 (PMC11287389; doi:10.1001/jamanetworkopen.2024.21846)
Supplement: Supplement 1. — eFigure. Correlation Between Serum Leptin Levels and BMI eTable 1. Details of Counties and States of Retrospective Cohort (2017-2022) eTable 2. Characteristics of Prospectively Enrolled Patients eTable 3. Serum Levels of Leptin in BC and Non-BC Patients [file jamanetwopen-e2421846-s001.pdf]

Supplementary Online Content

Sudan SK, Sharma A, Vikramdeo KS, et al. Obesity and risk of early onset and diagnosis of luminal A and triple-negative breast cancer subtypes in Black women: NIMHD Social Epigenomics Program. *JAMA Netw Open*. 2024;7(7):e2421846. doi:10.1001/jamanetworkopen.2024.21846

eFigure. Correlation Between Serum Leptin Levels and BMI
eTable 1. Details of Counties and States of Retrospective Cohort (2017-2022)
eTable 2. Characteristics of Prospectively Enrolled Patients
eTable 3. Serum Levels of Leptin in BC and Non-BC Patients

This supplementary material has been provided by the authors to give readers additional information about their work.

© 2024 Sudan SK et al. *JAMA Network Open.*

eFigure 1. Correlation between serum leptin levels and BMI.

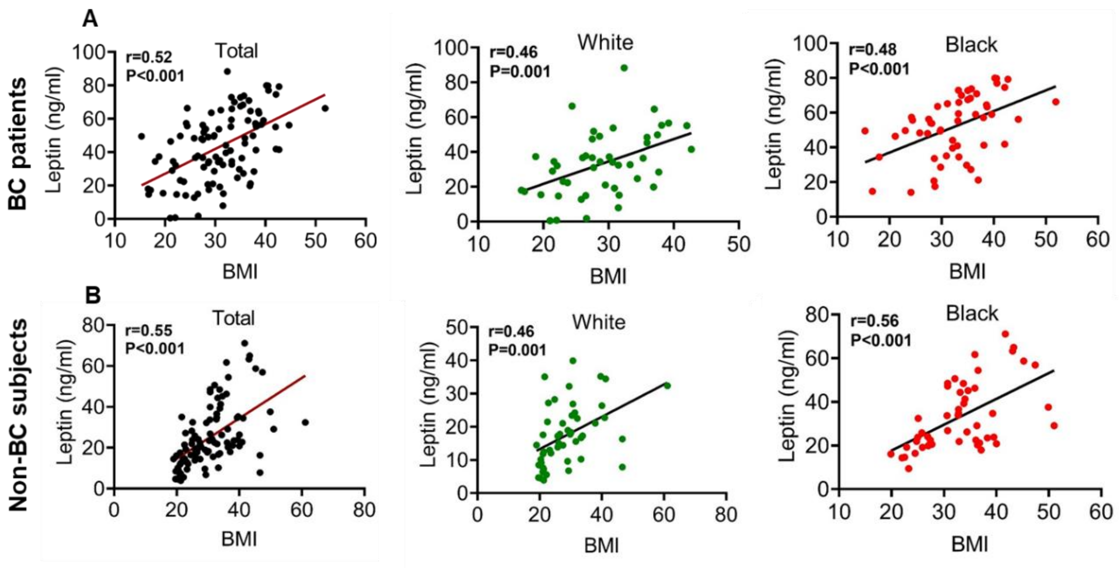

(A) Correlation between serum leptin and BMI of total, White, and Black BC and (B) non-BC subjects (P value=Pearson correlation).

© 2024 Sudan SK et al. *JAMA Network Open.*

eTable 1: Details of counties and states of retrospective cohort (2017-2022)

**County**	**State**	**Number of patients, No (%)**
Baldwin	AL	341 (31.43)
Cameron	LA	1 (0.09)
Cherokee	GA	1 (0.09)
Choctaw	AL	7 (0.65)
Clarke	AL	38 (3.50)
Clarke	MS	1 (0.09)
Coffee	AL	1 (0.09)
Covington	AL	3 (0.28)
Dale	AL	1 (0.09)
Dallas	AL	1 (0.09)
Escambia	AL	17 (1.57)
Escambia	FL	1 (0.09)
Forrest	MS	1 (0.09)
George	MS	16 (1.47)
Greenville	SC	1 (0.09)
Gwinnett	GA	1 (0.09)
Harrison	MS	14 (1.29)
Howell	MO	1 (0.09)
Jackson	MS	38 (3.50)
Jefferson	AL	1 (0.09)
Jones	MS	2 (0.18)
Lauderdale	MS	2 (0.18)
Lee	AL	1 (0.09)
Marengo	AL	2 (0.18)
Mobile	AL	542 (49.95)
Monroe	AL	9 (0.83)
Morgan	GA	1 (0.09)
Nash	NC	1 (0.09)
Okaloosa	FL	5 (0.46)
Ottawa	MI	1 (0.09)
Saint Joseph	MI	1 (0.09)
Santa Rosa	FL	3 (0.28)
Stone	MS	3 (0.28)
Washington	AL	20 (1.84)
Washington	FL	1 (0.09)
Wayne	MS	1 (0.09)
Whatcom	WA	2 (0.18)
Wilcox	AL	2 (0.18)
**Total**		1085

© 2024 Sudan SK et al. *JAMA Network Open.*

eTable2. Characteristics of prospective enrolled patients

		Number of patients, No. (%)			
		BC (n=99)		Non-BC (n=100)	
**Characteristic**					
Race		Black	White	Black	White
		53 (54)	46 (46)	50 (50)	50 (50)
Age	Median (y)	56	60	36	32
BMI	Normal weight (NW)	8 (15)	13 (28)	7 (14)	21 (42)
	Over weight (OW)	12 (23)	12 (26)	8 (16)	11 (22)
	Obese (Ob)	33 (62)	21 (46)	34 (68)	18 (36)
	Missing	0	0	1 (2)	0
Subtype	Luminal-A	29 (55)	33 (72)	NA	
	Luminal-B	2 (4)	4 (9)	NA	
	HER2-enriched	5 (9)	3 (6)	NA	
	TNBC	16 (30)	6 (13)	NA	
	Missing	1 (2)	0	NA	

Abbreviations: BC, Breast Cancer; BMI, body mass index (calculated as weight in kilograms divided by height in meters squared); y, years; TNBC, triple-negative breast cancer

© 2024 Sudan SK et al. *JAMA Network Open.*

eTable3: Serum levels of leptin in BC and non-BC patients

		Leptin (ng/ml)		
		**Black**	**White**	***P* value**
BC patients	Range (median)	14-80 (55.3)	0.5-89 (33.4)	*P* <0.001
	IQR	40.3-66.2	18.9-47.7	
Non-BC patients	Range (median)	9-72 (29.1)	3.5-40 (16.5)	*P* <0.001
	IQR	21.1-46.5	10.0-22.9	

© 2024 Sudan SK et al. *JAMA Network Open.*
