# Supplementary material for: Obesity and Early-Onset Breast Cancer and Specific Molecular Subtype Diagnosis in Black and White Women: NIMHD Social Epigenomics Program
Source: JAMA Netw Open. 2024 Jul 29;7(7):e2421846. doi: 10.1001/jamanetworkopen.2024.21846 (PMC11287389; doi:10.1001/jamanetworkopen.2024.21846)
Supplement: Supplement 2. — Data Sharing Statement [file jamanetwopen-e2421846-s002.pdf]

Data Sharing Statement

Sudan. Obesity and Risk of Early Onset and Diagnosis of Luminal A and Triple-Negative Breast Cancer Subtypes in Black Women. *JAMA Netw Open*. Published July 29, 2024. doi:10.1001/jamanetworkopen.2024.21846

Data
Data available: Yes
Data types: Deidentified participant data
How to access data: seemasingh@southalabama.edu
When available: With publication

Supporting Documents
Document types: None

Additional Information
Who can access the data: Researchers whose proposed use of the data has been approved
Types of analyses: For specified purpose
Mechanisms of data availability: With investigator support, after approval of a proposal, and with a signed data access agreement
Any additional restrictions: None
